# *Haemoproteus* parasites and passerines: the effect of local generalists on inferences of host–parasite co-phylogeny in the British Isles

**DOI:** 10.1017/S0031182023000628

**Published:** 2023-12

**Authors:** Charlie Woodrow, Adina Teodora Rosca, Rachel Fletcher, Abigail Hone, Marcello Ruta, Keith C. Hamer, Jenny Claire Dunn

**Affiliations:** 1 Joseph Banks Laboratories, School of Life Sciences, University of Lincoln, Green Lane, Lincoln LN6 7DL, UK; 2 School of Biology, University of Leeds, Clarendon Way, Leeds LS2 9JT, UK

**Keywords:** cospeciation, disease, *Haemoproteus*, mtDNA, phylogenetics

## Abstract

Host–parasite associations provide a benchmark for investigating evolutionary arms races and antagonistic coevolution. However, potential ecological mechanisms underlying such associations are difficult to unravel. In particular, local adaptations of hosts and/or parasites may hamper reliable inferences of host–parasite relationships and the specialist–generalist definitions of parasite lineages, making it problematic to understand such relationships on a global scale. Phylogenetic methods were used to investigate co-phylogenetic patterns between vector-borne parasites of the genus *Haemoproteus* and their passeriform hosts, to infer the ecological interactions of parasites and hosts that may have driven the evolution of both groups in a local geographic domain. As several *Haemoproteus* lineages were only detected once, and given the occurrence of a single extreme generalist, the effect of removing individual lineages on the co-phylogeny pattern was tested. When all lineages were included, and when all singly detected lineages were removed, there was no convincing evidence for host–parasite co-phylogeny. However, when only the generalist lineage was removed, strong support for co-phylogeny was indicated, and ecological interactions could be successfully inferred. This study exemplifies the importance of identifying locally abundant lineages when sampling host–parasite systems, to provide reliable insights into the precise mechanisms underlying host–parasite interactions.

## Graphical abstract

**Figure:**
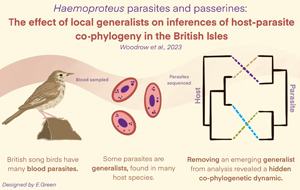

## Introduction

Throughout evolutionary history, ecological relationships between taxa have enabled the coevolution of distantly related organisms, resulting in an evolutionary trade-off between specialization to one niche with high efficiency or adaptation to several niches with lower efficiency (Brodie and Brodie, 1999). The first scenario may trigger arms races, the intensity of which can cause speciation to occur at the same rate in both groups of interacting organisms (Brodie and Brodie, 1999; Page, 2003), a phenomenon known as cospeciation. For studies of antagonistic cospeciation, and the driving forces of accelerated evolutionary rates predicted by the Red Queen hypothesis (Hamilton, 1980; Page, 2003), phylogenies of interacting parasites and hosts have long offered a testable model system (Page, 2003; Brockhurst and Koskella, 2013; de Vienne *et al*., 2013). In situations where parasites display strict host specificity through cospeciation, the dynamics of the host–parasite arms race can be explored to understand how constant change in the 2 groups allows them to coexist while overcoming each other's novel evolutionary adaptations (Van Valen, 1973; Hamilton, 1980). Cospeciation, however, is not simply synonymous with parasite specialization; host-switching, duplication, sorting events and inertia can also shape the cospeciation process. Host-switching occurs when a parasite successfully infects and adapts to a novel host, diverging from its original form, whereas duplication occurs when a parasite diverges and speciates within its original host (Filipiak, 2016). Sorting events occur when a parasite becomes extinct within a host species, and inertia occurs when hosts speciate but the parasite fails to diverge (Filipiak, 2016).

If host–parasite interactions were determined solely by cospeciation, then the phylogenetic branching patterns and timing of lineage divergence in the parasite would match those of the host (Fahrenholz's rule; Brooks, 1979). Based upon this rule, it is possible to apply cost-based analyses to comparisons of topological distances between parasite and host phylogenies, allowing us to investigate additional ecological mechanisms affecting the host–parasite relationship, including host switching, independent speciation and extinction (Page, 2003; Ricklefs *et al*., 2004; de Vienne *et al*., 2013). Coevolutionary analyses work exclusively on the assumption that congruent host–parasite phylogenies suggest cospeciation, while incongruent phylogenies suggest alternate mechanisms (Brooks *et al*., 1991; Page, 2003). Additionally, host–parasite interactions are a powerful system for investigating both molecular and macroevolutionary relationships, as well as for investigating local adaptation (Page, 2003). Despite this, few studies have attempted to investigate how parasites diverge with their hosts within restricted geographic domains.

Malaria and malaria-like parasites are an excellent model for inferring both ecological and coevolutionary relationships due to the presence of malarial parasites on all continents except on Antarctica (Valkiūnas, 2005), and their adaptation to a diverse range of vertebrate hosts (Lauron *et al*., 2015). In both mammals and birds, there is evidence of cospeciation of haemosporidian blood parasites (Ricklefs and Fallon, 2002; Garamszegi, 2009), including the causative agents of malaria (*Plasmodium* spp.; Lauron *et al*., 2015). In particular, the interactions between *Plasmodium* spp. and malaria-like *Haemoproteus* spp. and their avian hosts provide a key system for investigating the coevolution of hosts and their parasites (Waldenström *et al*., 2004).

Traditional studies have found a high level of host specificity of *Haemoproteus* spp. with avian hosts (Bennett *et al*., 1994), suggesting coevolution by cospeciation. However, recent contributions have found that while cospeciation can occur, host switching and parasite extinction are more potent drivers of *Haemoproteus* diversification (Ricklefs *et al*., 2004; Ciloglu *et al*., 2020). Individual parasite lineages and local adaptations vary to such an extent that global cospeciation may not be prevalent; instead, different regions may display unique levels of cospeciation and host switching, based on the localized prevalence of lineages and diversity of hosts (Forbes *et al*., 2002). Phylogenies built from mitochondrial cytochrome-*b* sequences have shown that *Haemoproteus* lineages are able to move between hosts of different genera or even different families, depending on the presence and diversity of available hosts (Bensch *et al*., 2000).

Here, we assess the extent of cospeciation and host-switching between *Haemoproteus* parasites and passerine hosts from the British Isles. We selected avian haemosporidians as the model system because of their sheer diversity and widespread distribution, which may have facilitated high host–parasite cospeciation and local adaptation. The British Isles are relatively under-sampled from an avian haemoparasite perspective, with only 13 *Haemoproteus* lineages from 6 host species previously recorded in the MalAvi database (accessed 04/01/21; Bensch *et al*., 2009). Here, we sample British passerines more extensively, with a focus on local inferences of co-phylogeny across all detected host–parasite associations, to test the relative importance of 5 potential drivers of co-phylogeny (cospeciation, host-switching, duplication, sorting events and inertia) using both distance-based and event-based methods. We then test the implications of removing both infrequently sampled lineages and generalist lineages, for our interpretation of the drivers of co-phylogeny.

## Materials and methods

### Sample sites

Bird blood samples were collected from sites in the east of the UK: in Essex and Norfolk (April–July 2014) and in Lincolnshire (2017 and 2018) as part of wider studies on passerine Haemosporidia prevalence. Three sites in Essex were near Tolleshunt D'Arcy (51°77′N, 0°79′E), Marks Tey (51°88′N, 0°79′E) and Silver End (51°85′N, 0°62′E); 1 site in Norfolk was near Hilgay (52°56′N, 0°39′E) (Dunn *et al*., 2018); 4 sites in Lincolnshire were near Potterhanworth (53°11′N, 0°25′W), Glentham (53°31′N, 0°22′W), Eagle (53°11′N, 0°41′W) and North Carlton (53°13′N, 0°31′W) (Parsa *et al*., 2023). At all sites, birds were caught using mist nets in the areas of scrub and woodland surrounded by arable farmland. Each bird was fitted with a unique British Trust for Ornithology metal ring. Under Home Office licence, a small blood sample (~50 *μ*L) was collected from each bird by venepuncture of the brachial vein, conveyed into a capillary tube, collected into an Eppendorf tube and stored at −20°C until DNA extraction.

### Parasite detection

DNA was extracted from blood using DNeasy blood and tissue kits (Qiagen, Manchester, UK) following the manufacturer's instructions. Samples were screened for the presence of haemosporidian parasites (*Plasmodium*, *Haemoproteus* and *Leucocytozoon*) using polymerase chain reactions (PCRs) with primers targeting fragments of the mtDNA cytochrome-*b* gene. Each sample was tested with 3 primer sets, each using the same forward primer (UNIVF 5′-CAYATAYTAAGAGAAYTATGGAG-3′) and a different reverse primer (UNIVR1: 5′-GCATTATATCWGGATGWGNTAATGG-3′; UNIVR2: 5′-ARAGGAGTARCATATCTATCWAC-3′; UNIVR3: 5′-ATAGAAAGMYAAGAAATACCATTC-3′) (Drovetski *et al*., 2014). Reactions were carried out in 10 *μ*L reaction volumes containing 5 *μ*L QIAGEN Mastermix PCR buffer (final concentration 3 mm MgCl_2_; Qiagen, Manchester, UK), 0.2 *μ*L each primer (10 mm), 3.6 *μ*L RNase-free water and 1 *μ*L template DNA. For positive and negative controls, the 1 *μ*L template DNA was replaced with 1 *μ*L an avian DNA sample with known infection, and 1 *μ*L RNase-free water, respectively. Controls were produced separately for each PCR run to confirm amplification had been successful and that contamination had not occurred. The PCR protocol involved denaturation at 95°C for 15 min followed by 42 cycles of denaturation at 94°C for 30 s, annealing for 30 s at primer-specific temperatures (UNIVR1: 54°C; UNIVR2: 52°C; UNIVR3: 53°C) and 45 s extension at 72°C, and a final terminal extension at 72°C for 10 min. PCR protocols were carried out using a BioRad T100 Thermal Cycler (BioRad, Hercules, CA, USA). PCR products were visualized on a 1% agarose gel stained with GelRed (Cambridge Bioscience, Cambridge, UK). Positive samples with low DNA content based on gel imaging were purified using QIAquick PCR purification kits following the manufacturer's instructions (Qiagen, Manchester, UK). All samples were then sent for bidirectional sequencing by Macrogen Europe (Amsterdam, Netherlands).

### Host and parasite sequence data

Sequences were analysed in MEGA X (Kumar *et al*., 2018) and identified using the BLAST algorithm (Zhang *et al*., 2000) and the avian haemosporidian database MalAvi 2.3.8 (Bensch *et al*., 2009). Lineages which differed from known lineages by at least 1 base pair were named by association to the MalAvi lineage with the closest matching sequence. Where multiple novel lineages were found to be similar (but not identical) to the same existing lineage, their similarities to the existing lineage have been marked with a number to denote different lineages (e.g. A-like1 would represent a new lineage with a similar sequence to lineage A, whereas A-like2 represents a separate lineage also similar to A). All sequences were then aligned using CLUSTAL W (Larkin *et al*., 2007) prior to phylogenetic analyses. Due to the unknown scale at which local host–parasite processes might operate, the data were checked for any evidence of spatially distinct host–parasite interactions between the groups of sites and found no strong evidence for differing dominant lineage composition (i.e. host species sampled in both locations did not differ in dominant parasite lineages), so merging data from sites could be justified. Sequence data of the mtDNA cytochrome-*b* gene from 23 British passerine species were collected based on GenBank (accession numbers provided in Appendix A) to construct the host phylogeny. Final alignments consisted of 708 bp for hosts, and 479 bp for parasites.

### Phylogenetic and statistical analyses

Bayesian phylogenetic reconstructions of parasites and hosts were generated using BEAST v1.10.4 (Papastamoulis *et al*., 2020). jModelTest v2.1.10 (Darriba *et al*., 2012; Suchard *et al*., 2018) determined that a generalized time reversible substitution model with Gamma distribution and a proportion of invariant sites was the best suitable nucleotide substitution model for both host and parasite alignments. Priors were defined using the BEAUTi v1.10.4 (Suchard *et al*., 2018) interface, including a strict clock and Yule speciation process for the parasite phylogeny (Galen and Witt, 2014) and an uncorrelated relaxed clock and Yule speciation process for the host phylogeny (Prum *et al*., 2015). Markov chain Monte Carlo simulations were run with chain length = 10 000 000 and 10% burn-in. Convergence of parameters and their effective sample size were checked in Tracer v1.7.1 (Rambaut *et al*., 2018) and a maximum clade credibility tree was computed in Tree Annotator v1.10.4 (Larkin *et al*., 2007).

### Co-phylogenetic analyses

To test for congruence between host and parasite phylogenies, we used both the ParaFit function in ‘ape’ (Legendre *et al*., 2002) and the PACo function in ‘paco’ (Hutchinson *et al*., 2017) in R 3.5.2 (R Core Team, 2021). These are both distance-based functions that test the null hypothesis that host and parasite phylogenies are independent from one another (i.e. the interacting groups speciated independently) and were favoured as they allow for different numbers of host and parasite terminal taxa. In ParaFit, we completed this analysis once for the full dataset, again with all rare (singly occurring) lineages removed, and then we conducted a sensitivity analysis by individually removing each parasite lineage in turn. Where parasites had only a single host, the host was also removed.

An event-based tree-reconciliation co-phylogenetic analysis was carried out using Jane 4.01 (Conow *et al*., 2010). We ran 11 different models with different cost schemes, as recommended by Benovics *et al*. (2020) and detailed in Table 1, in order to address a previously criticized disadvantage of cost-based analyses (de Vienne *et al*., 2013). We set a population size of 100, running for 500 generations. We then compared the total cost of each most parsimonious solution against 1000 random permutations of both the parasite phylogeny and host–parasite tip mapping to infer the likelihood that the phylogenies are likely to have arisen by chance, or suggest a co-phylogenetic relationship. This additional analysis was designed to assess the likelihood of differing ecological events being inferred from differing cost parameters.

**Table 1.** Outputs of co-phylogenetic analyses from Jane, using 11 models with different cost schemes

Model	Event costs	Total cost	Cospeciation	Duplication	Duplication and host switch	Sorting events (loss)	Inertia (failure to diverge)
Jane default*	0, 1, 2, 1, 1	86	5	9	14	39	10
TreeMap default*	0, 1, 1, 1, 1	72	5	8	15	39	10
TreeFitter default*	0, 0, 2, 1, 1	75	5	11	12	41	10
Codivergence-adjusted Treefitter model*	1, 0, 1, 1, 1	67	1	11	16	40	10
Host switch-adjusted TreeFitter model*	0, 0, 1, 1, 1	63	4	11	13	40	10
Cospeciation prohibitive*	10, 1, 1, 1, 1	79	0	6	22	41	10
Duplication prohibitive	1, 10, 10, 1, 1	263	8	8	12	45	10
Host switch prohibitive*	1, 1, 10, 1, 1	121	8	20	0	83	10
Sorting prohibitive*	1, 1, 1, 10, 1	428	2	8	18	39	10
FTD prohibitive*	1, 1, 1, 1, 10	167	2	8	18	39	10
Equal weights*	1, 1, 1, 1, 1	77	2	8	18	39	10

* Significant at *P* < 0.05.

### Host specificity indices

First, when investigating host–parasite relationships as local scales, we must consider whether the richness of parasite lineages results from the number of hosts sampled. Thus, we assessed the relationship between host sample size and parasite richness (number of lineages) using a general linear regression model in R 3.5.2 (R Core Team, 2021).

To classify parasite lineages as specialists or generalists, we use 2 measures of host specificity suggested by Poulin *et al*. (2011), both of which take into account the phylogenetic relatedness between multiple host species. The first examines phylogenetic distinctiveness among host species (SPD*_i_*), which is independent from the number of host species used by a parasite (Clarke and Warwick, 2001), using distance matrices. To implement this calculation we used the *taxondive* function in the *vegan* package (Oksanen *et al*., 2017) in R. The second index quantifies phylogenetic diversity of host species (PD*_i_*) using Faith's phylogenetic diversity, which incorporates host species richness into the phylogenetic diversity index (Faith, 1992). To implement this, we used the *pd* function in the *picante* package (Kembel *et al*., 2010) in R. For both indices, higher values represent more generalist parasite lineages. Both indices only function for parasite lineages found in 2 or more hosts, and thus were used to rank specificity in only the most generalist lineages. Parasite lineages which were found in only a single host species show maximum specificity and thus were considered specialists for discussion purposes.

## Results

**Figure 1.**
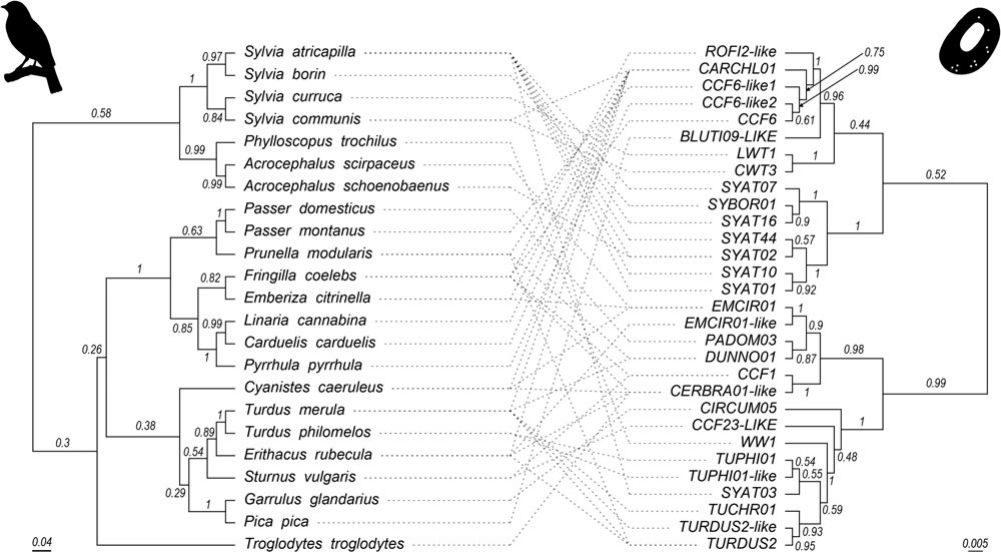
Tanglegram of passerine (left) and *Haemoproteus* (right) phylogenies with association lines and posterior probabilities. Details of phylogenetic reconstruction are provided in the ‘Materials and methods’ section.

As part of the aforementioned wider study, a total of 464 blood samples from 32 passerine species (Appendix A) were screened for haemosporidian infection (*Plasmodium*, *Haemoproteus* and *Leucocytozoon*), of which 268 (58%) tested positive for infection. For this analysis, 108 parasite sequences were isolated from 23 host species, representing 30 lineages of *Haemoproteus* (for details of host–parasite associations, see Fig. 1). Nine of these sequences were not present in the MalAvi database, and so represent new lineages. Twenty-three of the 30 *Haemoproteus* lineages were found in only 1 host species (specialist lineages) and of those, 17 were found in only 1 individual (rare lineages). The blackcap (*Sylvia atricapilla*) displayed the highest parasite richness and the highest parasite specificity with 7 different lineages, 6 of which were found exclusively in this species.

### Tests for co-phylogeny

**Figure 2.**
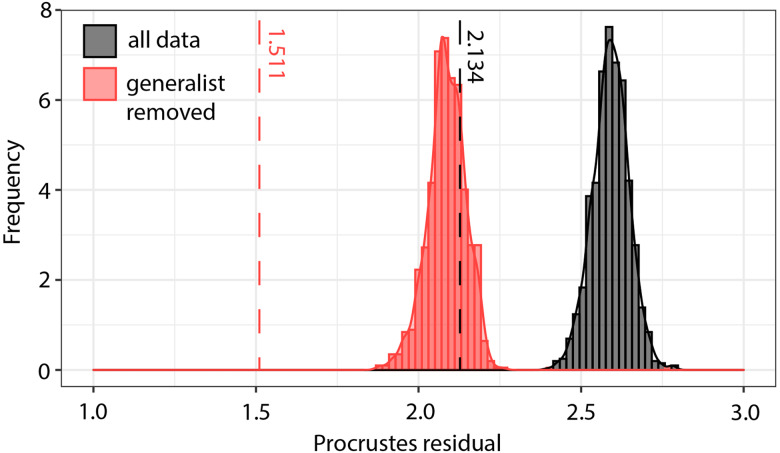
PACo analysis for host–parasite phylogenetic independence. The observed best-fit Procrustean super-impositions (vertical dotted lines) are lower than the randomized distributions (histograms) for both the full dataset (black), and the dataset when the generalist lineage is removed (red), indicating the co-evolutionary relationship between parasite and host is stronger than the same for any ensemble of network randomizations in the null model. However, when the generalist is removed, the association becomes stronger (lower Procrustes residual) in both the observed best-fit Procrustean super-impositions and the randomized distributions. Histogram bins = 100, smooth line through histograms = means.

**Figure 3.**
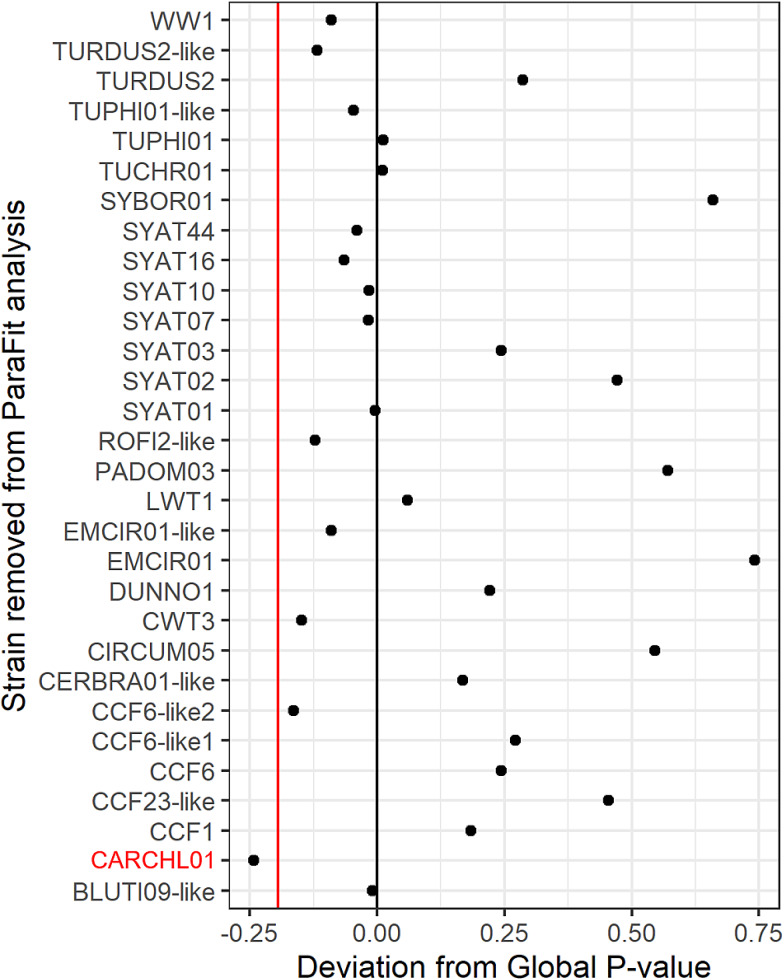
Sensitivity analysis, showing the effect of removing each individual parasite lineage on the ParaFit global *P* value. Black line at 0 represents the global *P* value when the full dataset is used (*P* = 0.245). Red line indicates the point at which the change in *P* value indicates a statistically significant coevolutionary inference (*P* < 0.05).

To test for co-phylogeny, the ParaFit function in the *ape* package (Legendre *et al*., 2002) and the PACo function in the *paco* package (Hutchinson *et al*., 2017) in R 3.5.2 (R Core Team, 2021) were used. When all lineages were used in analysis, the ParaFitGlobal test found no support for co-phylogeny (*P* = 0.245, based on 999 random permutations; *n* = 30 lineages). Additionally, this test found no support for co-phylogeny when all singly detected parasite lineages were removed from the dataset (*P* = 0.293, based on 999 random permutations; *n* = 13 lineages; Appendix B). However, when only the generalist parasite CARCHL01 was removed, the test found support for co-phylogeny (*P* = 0.003, based on 999 random permutations; *n* = 29 lineages). The PACo analysis revealed that the observed best-fit Procrustean super-impositions were lower than the randomized distributions for both the full dataset and the dataset when the generalist lineage was removed (Fig. 2). This indicates that the co-evolutionary relationship between parasite and host is stronger than the same for any ensemble of network randomizations in the null model, indicating co-phylogeny (Hutchinson *et al*., 2017). When the generalist is removed, the association becomes stronger (lower Procrustes residual) in both the observed best-fit Procrustean super-impositions and the randomized distributions, indicating that the generalist lineage is changing our inferences of the strength of the association. Sensitivity analysis revealed that removal of other parasites resulted in great variation in the global *P* value (Fig. 3), but only the removal of the generalist lineage produced a significant co-phylogenetic inference.

### Inferring ecological interactions

Once the generalist parasite had been removed from the dataset, the ecological interactions that may have occurred to facilitate coevolution of parasite and host lineages could be investigated. Event-based tree-reconciliation co-phylogenetic analyses were carried out using Jane 4.01 (Conow *et al*., 2010). Differing from ParaFit and PACo, Jane allows for cost-based inferences of the potential interactions between parasite and host lineages that could have facilitated coevolution. From the 11 different cost schemes applied for co-phylogenetic reconstructions in Jane, all the default models had equally high occurrences of duplication, host switch and loss events. The least cost models provided by Jane were the Treefitter model of co-phylogeny that was adjusted for host switching (cost = 63), and the codivergence-adjusted Treefitter model (cost = 67). Across all 11 models, duplication and host switching, and loss, seem to be the most important drivers of parasite speciation. The duplication-prohibited, host-switch prohibitive and sorting-prohibited models resulted in high numbers of loss events and represented scenarios with the greatest costs; supporting inferences of host switching and loss as major parasite speciation mechanisms in this avian haemosporidian system. However, the duplication-prohibited model was the only scenario whereby global costs were not statistically significant. Across the other models, loss was almost always the most important factor in the model, followed by host-switching. The only exception to this was the failure to diverse prohibitive model, whereby the high cost of failure to diverge outweighed the contributions of loss or host-switching. In the model assuming equal weights of the costs of each cospeciation event, loss remained the most important factor, followed by host-switching and failure to diverge.

### Host specificity indices

Both measures of host phylogenetic distinctiveness and host phylogenetic diversity confirmed that the most generalist lineage in our dataset was CARCHL01, with both indices for CARCHL01 nearly double that of the next most generalist lineage (Table 2). Comparison between preliminary host and parasite phylogenies (Fig. 1) indicated the presence of a single highly generalist lineage, CARCHL01, present in 8 different host species. There was also no observed relationship between parasite richness and host sample size (*t* = 0.312, *P* = 0.758).

**Table 2.** Measures of phylogenetic distinctiveness (SPD*_i_*) and phylogenetic diversity (PD*_i_*) for all parasite lineages with more than 1 host species (Poulin *et al*., 2011)

Parasite lineage	No. of host species	Total no. of hosts	SPD*_i_*	PD*_i_*
CARCHL01	8	12	2.90	1.80
CCF1	2	2	0.78	0.47
CERBRA01-like	4	8	1.51	0.94
DUNNO01	2	19	0.75	0.47
EMCIR01	3	13	1.15	0.71
SYAT02	2	10	0.77	0.46
TURDUS2	3	11	1.11	0.71

Higher values of both metrics represent more generalist lineages.

## Discussion

High degrees of host–parasite specificity are hallmarks of coevolutionary relationships (Ricklefs and Fallon, 2002). In this study, this specificity was initially suggested by a high proportion of unique lineages, but a global test of co-phylogeny using ParaFit and PACo on both the full dataset and the dataset excluding rare lineages suggested that passerine and *Haemoproteus* phylogenies are independent from one another. However, when the most generalist lineage identified by the parasite specificity index (CARCHL01) was removed from the dataset, the analysis revealed a stronger co-phylogenetic relationship between parasites and hosts. This effect was not retrieved following the removal of any other parasite lineage, indicating the generalist was masking the relationship. Event-cost-based analysis suggested that once the generalist is removed, the host and parasite lineages display a co-phylogenetic relationship, because the costs of the association were significantly lower in the observed data than in the random permutations of both the parasite tree and the host–parasite association mapping. This analysis also suggested that cospeciation is not a common mode of parasite proliferation in this system. Instead, it is the duplication of parasite lineages and the switching between hosts which has facilitated the coevolution of these groups. Failure of the parasite lineages to diverge between hosts on multiple occasions, as suggested by cost-based analyses, also support reduced cospeciation in this system (Hamilton, 1980; Brockhurst and Koskella, 2013). Altering the cost of cospeciation had no great effect on the results, suggesting that the cost-based method is more reliable for inferring parasite proliferation events in the history of a host–parasite association than previously assumed (de Vienne *et al*., 2013). These results do not support previous findings that cospeciation is a common mode of parasite proliferation in an avian Haemosporidia system (Ricklefs and Fallon, 2002), but instead agree with more recent contributions that host-switching and a lack of divergence of parasites is responsible for the observed relationships.

These data also support the hypothesis that different regions display unique levels of co-phylogeny, or local adaptation (Forbes *et al*., 2002; Ricklefs *et al*., 2004). Sensitivity analyses reveal that inferences of local parasite–host co-phylogeny can easily change with the level of sampling of the local system, with greatly varied global *P* values when any single parasite lineage is removed (Fig. 3). This idea of local specificity is also exemplified in the finding of a *Haemoproteus* parasite in a wren (*Troglodytes troglodytes*), of which the MalAvi database reports only 2 examples (Bensch *et al*., 2009; Ellis *et al*., 2020). It is possible either that such low frequencies of this parasite may be in circulation that detection is very low as in this case; or that locally, this parasite has adapted to infect a higher proportion of its hosts. Either way, with any analyses of this kind, many rare lineages, potentially unique to the local system, may be present yet undiscovered. Such rare lineages must be uncovered in local systems to more accurately represent any co-phylogenetic inference. Potential local adaptation may further be influenced by factors such as variation in anthropogenic food provisioning, by which placement of rural bird feeders results in variation in the proximity and density of heterospecific hosts (Moyers *et al*., 2018). This may alter the likelihood of parasite transmission and proliferation of dominant lineages, due to the frequent placement of feeders near areas of scrub and stagnant water (J. C. Dunn, personal observation); ideal for the developmental needs of the vectors of haemosporidian parasites (Conte *et al*., 2007).

This idea of differing heterospecific compositions influencing host–parasite systems is also supported by the result that parasites proliferate in this system by host switching, perhaps as a product of increased heterogeneous vector-feeding tendencies resulting from increased interactions with novel hosts (Lauron *et al*., 2015). The UK is home to over 30 species of mosquito (Snow, 1990), and there remains limited evidence as to which parasite lineages different mosquito species are able to successfully transmit (Dunn, in prep). Other vectors, such as biting midges (*Culicoides* spp.) and flatflies (Hippoboscidae), of which even less is known, are linked to *Haemoproteus* transmission (Valkiūnas, 2005) and there is a general lack of knowledge of these transmission networks (Pérez-Tris *et al*., 2007). These vector species are also coevolving with parasites and avian hosts through their role as the definitive host, and will thus drive many aspects of parasite/host life histories (Hurd, 2003). Additionally, vectors can transfer parasite sporozoites into the bloodstream of avian hosts that may not be suitable for proliferation of a transmissible infection. As PCR analysis is able to pick up the DNA of these sporozoites (Valkiūnas *et al*., 2009), it is not often possible to confirm competent hosts for parasite lineages without microscopy. Although this limits the potential inferences of a purely molecular investigation, the presence of CARCHL01 gametocytes in all detected host species in this study has been confirmed through microscopy, to rule out PCR contamination as an answer to the generalist definition of CARCHL01 (Armour *et al*., in prep).

The high degree of blackcap parasite endemism is not unique to these data. Previous findings suggest that sympatric speciation within the blackcap is occurring as a result of ecological variation between parasite lineages, such as transmission rates and vector specificity (Bensch *et al*., 2004; Pérez-Tris *et al*., 2007). These differences in ecology and life histories have allowed for differential exploitation of the host, allowing for speciation without host switching (Schluter, 2000; Pérez-Tris *et al*., 2007), and supports the hypothesis of interspecific variation in parasite diversification rates. Additionally, genetic distance matrices using cytochrome-*b* have suggested that cytochrome-*b* nucleotide divergence occurs around 3 times slower in parasites than in hosts (Ricklefs and Fallon, 2002; de Vienne *et al*., 2013), which would strengthen the hypothesis that it is more efficient for the parasite to proliferate by mechanisms other than cospeciation, as parasite and host rates of evolution are not temporally homogenous (Hafner *et al*., 1994; Page *et al*., 1998; Ricklefs *et al*., 2004). It has also been found that parasite clades with mtDNA sequence divergence as little as 0.5% may be found in different hosts (Ricklefs *et al*., 2004), further suggesting that the most common mode of inter-host parasite proliferation may be host switching rather than cospeciation. Alternatively, one may argue that these data provide an observation of a cryptic population dynamic, in which rapid evolution of the parasite is masking the true host–parasite interaction (Yoshida *et al*., 2007). This is plausible given that CARCHL01 was not observed in 2014–2017 (from *n* = 43 sequences) but constituted 18% of sequences in 2018 (*n* = 65). It could also be that the potential emergence of this generalist was detectable at more of a local level than the British Isles, as it was present at the Lincolnshire sites but not at the Essex/Norfolk sites. Although in other regions CARCHL01 is thought to be a finch specialist, the data suggest it to be a local generalist, supporting the observation that parasite lineages can be found in different host species in different locations [e.g. DUNNO01 found in *Emberiza citrinella* elsewhere in the UK (Dunn *et al*., 2014); but not found in *E. citrinella* in this study, despite being highly prevalent in *Prunella modularis*].

It is important to note that the data collected in this study will be only a subset of local *Haemoproteus* lineages and passerine species, and further sampling may refine the coevolutionary dynamic (Pérez-Tris *et al*., 2007). Although this could be used to explain the high blackcap parasite endemism, as we sampled from 41 blackcaps, we sampled from similar numbers of blue tit (42), dunnock (37), whitethroat (35) and blackbird (37), but did not observe a similar level of parasite endemism. Similarly, we sampled only 1 garden warbler (*Sylvia borin*), but this provided a unique lineage. We also found that host sample size does not display any statistically significant relationship with parasite richness, thus we believe that screening for such endemic parasite lineages at local spatial ranges is a viable method for the future studies. However, the frequencies of inferred evolutionary events such as cospeciation and host-switching are also likely to display variation depending on the level of sampling. Therefore, it would be helpful to conduct new investigations by further developing the local dataset, and to collect further information on the environmental characters which could influence parasite transmission. Comparing this local pattern to others gleaned from other regions will provide a circumspect evaluation of the influence that biogeographical factors, environmental conditions (Wolinska and King, 2009; Drovetski *et al*., 2014), anthropogenic food provisioning or nesting densities exert on local parasite prevalence. The monitoring of the dynamics of parasite–host associations over time, and the variation in such associations at the local level, is vital to understanding the specialist–generalist definitions of parasite lineages, and unmasking hidden coevolutionary dynamics.

## Data Availability

DNA sequences created in this study are available at GenBank (accession codes MT299243–MT299288). GenBank accessions for the sequences used to create the host phylogenetic tree are provided in Appendix A. R code for ParaFit test available upon request, but also freely available in the R CRAN repository under the package ‘Ape’.

## Appendix A

**Table:** Host sample sizes used in study (not including species which did not pick up a Haemosporidia infection), and GenBank accession codes for host sequence data of the mtDNA cytochrome-*b* gene used for analyses

Species	Accession code	Sample size	Lineages found
*Acrocephalus schoenobaenus*	Z72475.1	4	1
*Emberiza citrinella*	EU571277.1	22	2
*Fringilla coelebs*	L76610.1	31	4
*Linaria cannabina*	KY378727.1	6	1
*Cyanistes caeruleus*	AF347961.1	42	3
*Passer domesticus*	AY495393.1	6	1
*Passer montanus*	D32170.1	5	1
*Phylloscopus trochilus*	DQ174598.1	5	1
*Pica pica*	HM185388.1	1	1
*Prunella modularis*	AY228080.1	37	3
*Pyrrhula pyrrhula*	KY378774.1	2	1
*Sturnus vulgaris*	AF285790.1	7	2
*Sylvia atricapilla*	AY308735.1	41	7
*Sylvia borin*	AJ534549.1	1	1
*Sylvia curruca*	AJ534536.1	3	1
*Sylvia communis*	AJ534538.1	35	2
*Troglodytes troglodytes*	AY156507.1	18	1
*Erithacus rubecula*	L78807.1	33	4
*Turdus merula*	DQ263757.1	37	5
*Turdus philomelos*	AY495411.1	8	2
*Garrulus glandarius*	U86034.1	3	1
*Acrocephalus scirpaceus*	KF614612.1	21	1
*Carduelis carduelis*	EU571279.1	18	1

## Appendix B

**Table:** Individual host–parasite associations and their contributions to the ParaFit *P* value before and after removal of CARCHL01

Host	Parasite	*P* value contribution	*P* value contribution with CARCHL01 removed
*T. merula*	CARCHL01	0.851	N/A
	DUNNO01	0.767	0.005**
	TUCHR01^†^	0.52	0.002**
	TURDUS2	0.613	0.004**
	TURDUS2-like^†^	0.402	0.004**
*S. atricapilla*	SYAT01	0.385	0.002**
	SYAT02	0.37	0.001***
	SYAT03^†^	0.626	0.002**
	SYAT07^†^	0.464	0.003**
	SYAT10^†^	0.38	0.001***
	SYAT16^†^	0.211	0.003**
	SYAT44^†^	0.361	0.001***
*C. caeruleus*	CARCHL01	0.2	N/A
	CERBRA01-like	0.125	0.001***
	EMCIR01	0.105	0.024*
*P. pyrrhula*	CCF6-like2^†^	0.032*	0.045*
*F. coelebs*	CCF1	0.51	0.055
	CCF6	0.63	0.4
	CERBRA01-like	0.629	0.61
	ROFI2-like^†^	0.002**	0.001***
*P. modularis*	CARCHL01	0.237	N/A
	DUNNO01	0.119	0.024*
	TURDUS2	0.17	0.058
*S. borin*	SYBOR01^†^	0.217	0.022*
*C. carduelis*	CARCHL01	0.973	N/A
*P. domesticus*	PADOM03^†^	0.508	0.485
*G. glandarius*	CIRCUM05^†^	0.405	0.049*
*S. curruca*	LWT1^†^	0.014*	0.01*
*L. cannabina*	CARCHL01	0.965	N/A
*P. pica*	BLUTI09-like^†^	0.024*	0.0171*
*A. scirpaceus*	EMCIR01	0.057	0.06
*E. rubecula*	CARCHL01	0.73	N/A
	CERBRA01-like	0.561	0.524
	EMCIR01-like^†^	0.01**	0.001***
	TURDUS2	0.475	0.025*
*A. schoenobaenus*	SYAT02	0.914	0.584
*T. philomelos*	TUPHI01	0.949	0.076
	TUPHI01-like^†^	0.045*	0.042*
*S. vulgaris*	CCF1	0.105	0.007**
	CCF23-like^†^	0.068	0.002**
*P. montanus*	CCF6-like1^†^	0.62	0.01**
*S. communis*	CARCHL01	0.974	N/A
	CWT3^†^	0.073	0.072
*P. trochilus*	WW1^†^	0.056	0.053
*T. troglodytes*	CERBRA01-like	0.58	0.45
*E. citrinella*	CARCHL01	0.221	N/A
	EMCIR01	0.103	0.1

^†^Rare parasite lineage.

**P* < 0.05, ***P* < 0.01, ****P* < 0.001.
